# Features of Autosomal Recessive Alport Syndrome: A Systematic Review

**DOI:** 10.3390/jcm8020178

**Published:** 2019-02-03

**Authors:** Jiwon M. Lee, Kandai Nozu, Dae Eun Choi, Hee Gyung Kang, II-Soo Ha, Hae II Cheong

**Affiliations:** 1 Department of Pediatrics, Chungnam National University Hospital, Daejeon 30515, Korea; jwmleemd@gmail.com; 2 Department of Pediatrics, Kobe University Graduate School of Medicine, Kobe 650-0017, Japan; nozu@med.kobe-u.ac.jp; 3 Department of Internal Medicine, Chungnam National University Hospital, Daejeon 30515, Korea; daenii@cnu.ac.kr; 4 Department of Pediatrics, Seoul National University Children’s Hospital, Seoul 03080, Korea; kanghg1@gmail.com (H.G.K.); ilsooha@snu.ac.kr (I.-S.H.); 5 Kidney Research Institute, Medical Research Center, Seoul National University College of Medicine, Seoul 03080, Korea

**Keywords:** Alport syndrome, autosomal recessive inheritance, systematic review, *COL4A3* gene, COL4A4 gene, mutation

## Abstract

Alport syndrome (AS) is one of the most frequent hereditary nephritis leading to end-stage renal disease (ESRD). Although X-linked (XLAS) inheritance is the most common form, cases with autosomal recessive inheritance with mutations in *COL4A3* or *COL4A4* are being increasingly recognized. A systematic review was conducted on autosomal recessive Alport syndrome (ARAS). Electronic databases were searched using related terms (until Oct 10th, 2018). From 1601 articles searched, there were 26 eligible studies with 148 patients. Female and male patients were equally affected. About 62% of patients had ESRD, 64% had sensorineural hearing loss (SNHL) and 17% had ocular manifestation. The median at onset was 2.5 years for hematuria (HU), 21 years for ESRD, and 13 years for SNHL. Patients without missense mutations had more severe outcomes at earlier ages, while those who had one or two missense mutations had delayed onset and lower prevalence of extrarenal manifestations. Of 49 patients with kidney biopsy available for electron microscopy (EM) pathology, 42 (86%) had typical glomerular basement membrane (GBM) changes, while 5 (10%) patients showed GBM thinning only. SNHL developed earlier than previously reported. There was a genotype phenotype correlation according to the number of missense mutations. Patients with missense mutations had delayed onset of hematuria, ESRD, and SNHL and lower prevalence of extrarenal manifestations.

## 1. Introduction 

Alport syndrome (AS) is a progressive hereditary nephritis leading to end-stage renal disease (ESRD) with sensorineural hearing loss and ocular abnormalities (OT), such as lenticonus or retinal flecks. X-linked AS (XLAS) caused by *COL4A5* mutations is the most common form, accounting for 80% of AS [1]. In recent years, with the help of genetic research techniques enabling facilitated screening of multiple genes, cases with autosomal AS caused by *COL4A3* or *COL4A4* mutations are being increasingly recognized. Autosomal dominant (ADAS, OMIM 104200) and autosomal recessive (ARAS, OMIM 203780) forms are reported to account for 5% and 15% of AS, respectively [1,2]. Since the cases are still rare and a portion of these patients may not have the typical presentation of AS, prompt diagnosis based on early recognition is essential to provide timely intervention with anti-proteinuric agents, such as angiotensin converting enzyme inhibitors (ACEI). We thereby sought to systematically review the clinical features and investigated the genotype phenotype correlation of patients with ARAS to help in understanding and managing this relatively infrequent disease.

## 2. Methods

### 2.1. Search Strategy and Data Extraction

We performed a PubMed and EMBASE search to identify eligible articles. Furthermore, a forward search of the retrieved articles was performed and “google scholar” was assessed to screen for non-indexed publications. The last search was performed on October 10th, 2018. 

**Figure 1:**
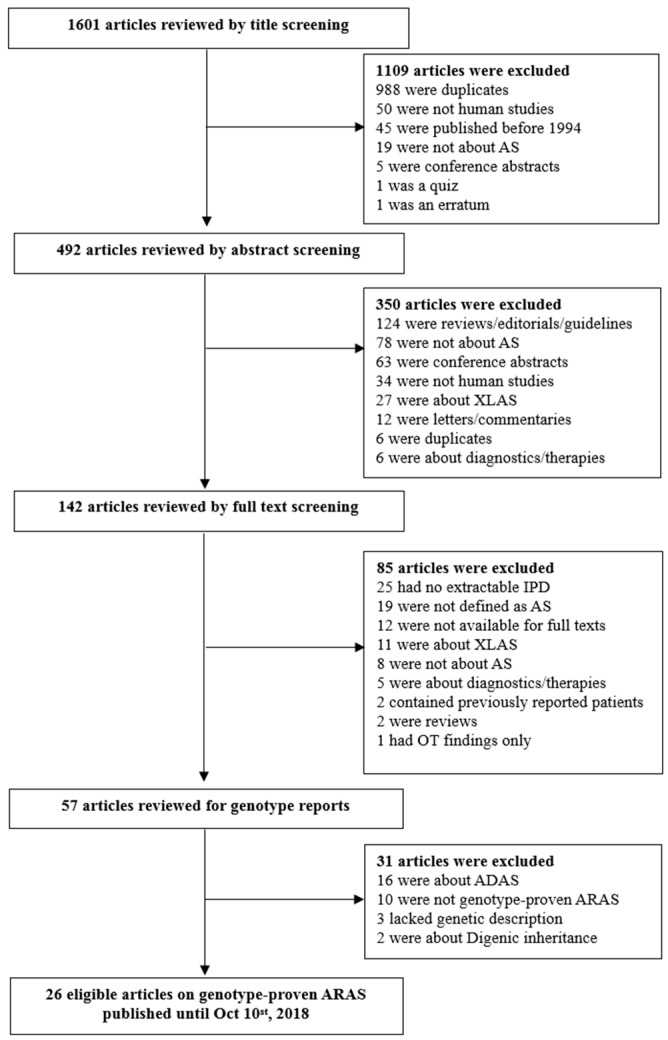
Flow chart of Literature search. AS, Alport syndrome; XLAS, X-linked AS; ADAS, autosomal dominant AS; ARAS, autosomal recessive Alport syndrome; IPD, individual patient data.

The search terms included: “Alport AND autosomal”, “Alport AND *COL4A3*”, and “Alport AND *COL4A4*”. We examined and screened the articles first by the titles, followed by the abstracts, and eventually by examining the full texts. The detailed process of article selection is presented in Figure 1. Data were extracted from all of the cases where genotypes were identifiable. Demographic information included age, gender, and ethnicity. Clinical manifestation included presence of renal and extrarenal manifestations and pathology reports. Information regarding sensorineural hearing loss (SNHL) and ocular abnormalities were collected from the clinicians’ reports. This report adhered to the Preferred Reporting Items for Systematic Reviews and Meta-analyses (PRISMA) guidelines (Supplementary Table S1) [3].

### 2.2. Selection of Studies

Two reviewers (Jiwon M. Lee and Kandai Nozu) working independently considered the potential eligibility of each abstract and title that resulted from the initial search. The full-text versions of the eligible studies were reviewed and discussed. Disagreements were harmonized by consensus or, if not possible, through arbitration by a third reviewer (Hae Il Cheong).

### 2.3. Eligibility and Exclusion Criteria

Duplicates, letters, commentaries, and replies were excluded. Because we used rather broad search terms in order not to leave out relevant studies, the initial search contained many duplicated papers. Original articles not containing patient data, such as review articles, were also excluded. Articles containing overlapping patients from previous works were excluded.

Although all cases from the literature were included as possible, we had certain eligibility criteria. First, the diagnosis of AS followed the expert guidelines by Savige et al. [4]: Diagnosis of AS was confirmed with the demonstration of a lamellated glomerular basement membrane (GBM), or two *COL4A3* or *COL4A4* mutations [4]. We therefore collected cases which had genotype proven compound heterozygous (ch) or homozygous (H) mutations in *COL4A3* or *COL4A4*, and excluded cases with single heterozygous mutation. Second, we compared the genotype results with public databases: HGMD^®^ Professional 2018.3 (https://portal.biobase-international.com/hgmd/pro/start.php), LOVD v.2.0 (http://www.lovd.nl/2.0/docs/index.php), and the 1000 Genomes Project data (http://www.internationalgenome.org/1000-genomes-browsers/). We thereby excluded cases with unknown pathogenicity. Third, articles published before 1994 were excluded because the autosomal form of AS was first reported in 1994 by Lemmink et al. [5] and Mochizuki et al. [6].

### 2.4. Statistical Analysis

Statistical analyses were performed using SPSS (IBM SPSS Statistics for Windows, version 20; IBM Corp., Armonk, NY, USA). Mann Whitney U tests, Pearson’s chi-squared tests (*χ*^2^), and Fisher’s exact tests were used as appropriate. Graphs of the occurrence of events (age at ESRD, age at detection of SNHL) were computed according to the Kaplan–Meier method, as in the study by Oka et al. [7].

## 3. Results

### 3.1. Study Selection and Characteristics

In this study, patients who had compound heterozygous or homozygous mutations with proven pathogenicity in either *COL4A3* or *COL4A4* were included.

We were initially able to identify 1601 articles using electronic and manual research. After reviewing titles and abstracts, 142 studies were selected for full-text reading. Of them, 85 were excluded due to irrelevance, or inappropriateness; there were 25 studies lacking individual data and 15 of them provided some grouped data available for aggregate patient data (APD) analysis [8,9,10,11,12,13,14,15,16,17,18,19,20,21,22]. Since these 15 studies lacked individual patient data and genotypes, they were excluded from the systematic review and their findings are discussed in discussion section of this study.

The remaining 57 studies were subject to genotype reading, which excluded 21 additional cases; 16 were about ADAS, 2 had digenic inheritance, 3 lacked genotype description. Of the remaining 36 studies, 10 were not genotype proven, or diagnosed by linkage analysis only [21], or had unknown pathogenicity.

Therefore, this analysis finally included 26 eligible articles with 148 patients (Figure 1) [7,23,24,25,26,27,28,29,30,31,32,33,34,35,36,37,38,39,40,41,42,43,44,45,46,47,48]. The respective characteristics of included studies are summarized in Table 1. The PRISMA checklist for systematic review is shown in Supplementary Table S1.

### 3.2. Clinical Features

There were 71 male and 74 female patients in this study. Patients were mostly of Caucasian and Asian ethnicity (Table 2). About 77% had affected family members and 30% had consanguinity in the family.

Hematuria (HU) was detected in all 93 cases with available information. The median ages at detection of hematuria and proteinuria (PU) age were 2.8 years and 6.5 years, respectively. In addition, 27/57 (47%) of patients had proteinuria of nephrotic range. Genetic diagnosis of ARAS was made at 20.0 median years. By the time of analysis, where median age of the patients at last follow-up (F-U) was 27 years, 62% of patients developed ESRD at median age of 21 years. Out of 129 patients with available data, 82 (64%) had SNHL at 13 median years. Ocular abnormality (OT) was reported in 15 out of 88 patients (17%), including 5 anterior lenticonus and 4 bilateral anterior subcapsular cataracts. The median age at onset of ocular manifestation was 32 years. However, this information on age was available only in two cases [37,47].

Of 49 patients with kidney biopsy available for electron microscopy (EM) pathology, 42 (86%) had typical glomerular basement membrane (GBM) changes of AS, such as lamellation, splitting, and irregularly wrinkled and/or basket-woven distortion (Table 3). Five (10%) patients initially had pathology features of diffuse thinning of the GBM only, thin basement membrane disease (TBMD). At light microscopy (LM), 7/49 (14%) patients showed pathology of focal segmental glomerulosclerosis (FSGS). Collagen α stain results were available in 34 patients and 26/34 (76%) patients had absent or abnormal α5 stains. Of those patients (8/34, 34%) who showed normal α5 expression pattern, 7 (88%) had at least one missense mutation (Table 3). 

### 3.3. Genotypephenotype Correlations

Out of 148 patients with compound heterozygous or homozygous mutations with proven pathogenicity, 96 had mutations in *COL4A3* and 52 in *COL4A4* (Table 4). Sixty-nine (46%) patients had no missense mutation, and 79 (53%) patients had one (31 patients, 21%) or two (48 patients, 32%) missense mutations. (Table 4). We grouped the patients according to their number of missense mutations in order to investigate genotype phenotype correlation.

Gender ratio remained 1:1 in all subgroups. There were more patients of Asian ethnicity in the group with missense mutation. Patients without missense mutations tended to be significantly more from consanguineous families compared to those with missense mutations (47% vs. 15%, *p* < 0.001) (Table 4). The group without missense mutations had earlier onset ages of hematuria, ESRD, and SNHL compared to missense group (2.0 vs. 5.6 years, *p* = 0.004; 21 vs. 26 years, *p* = 0.006; 6 vs. 18 years, *p* = 0.019; respectively). Patients without missense mutations also had higher prevalence of SNHL (87% vs. 42%, *p* < 0.001) and ocular abnormalities (29% vs. 6%, *p* = 0.006). Moreover, compared to patients who had 0 missense mutations, the 2-missense group had delayed onset of hematuria (10.5 vs. 2.0 years, *p* = 0.005), proteinuria (20.0 vs. 3.8 years, *p* = 0.044), ESRD (30 vs. 19 years, *p* = 0.005), and less prevalence of SNHL (*p* < 0.001) and ocular abnormality (*p* = 0.012) (Table 4).

**Figure 2:**
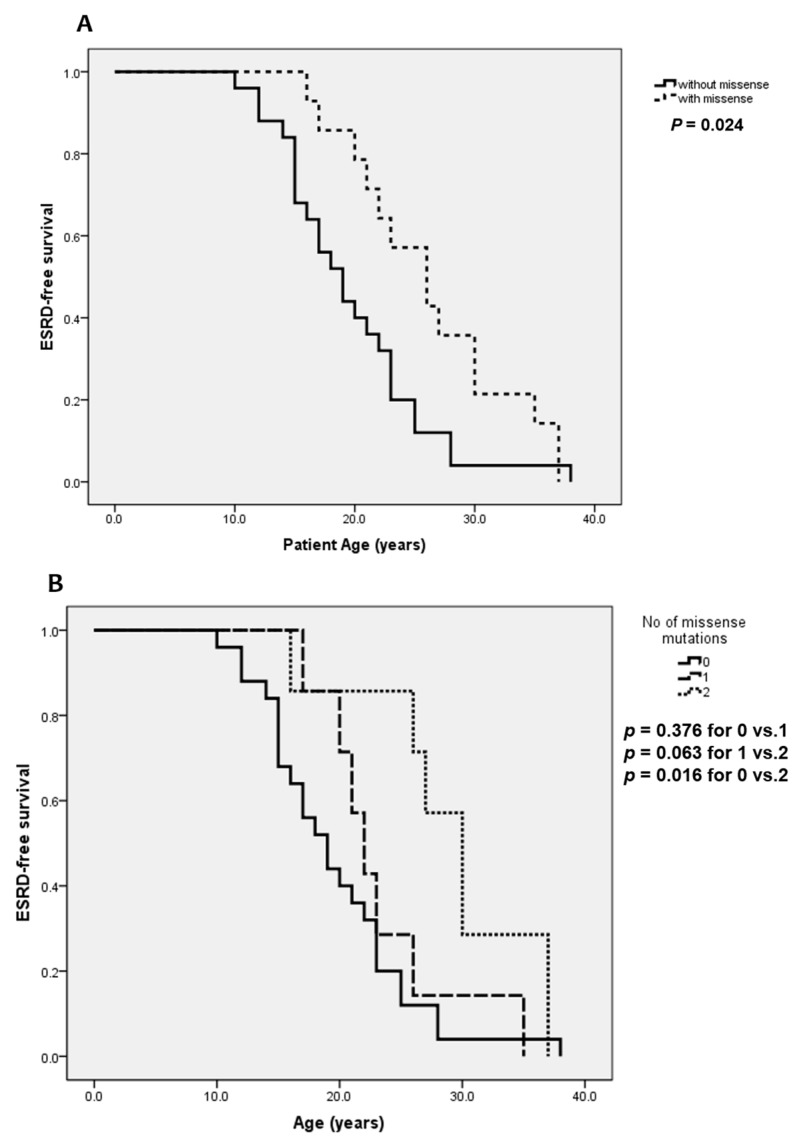
ESRD-free survival of ARAS patients according to (**A**) presence of missense mutation and (**B**) number of missense mutations.

**Figure 3:**
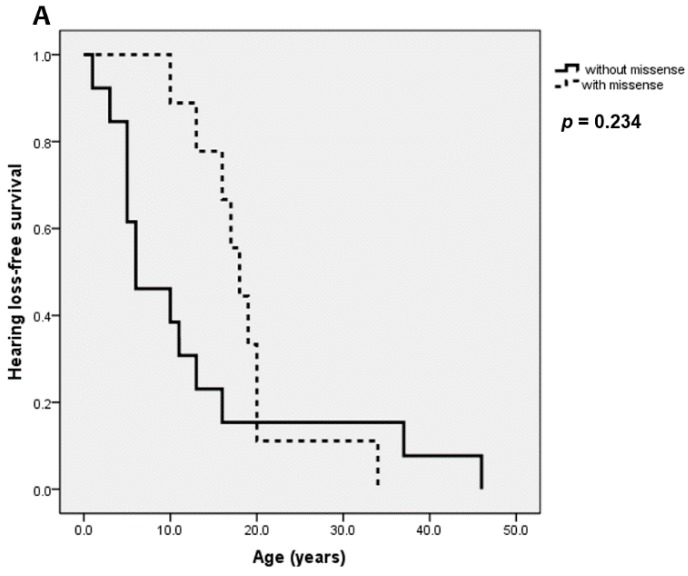

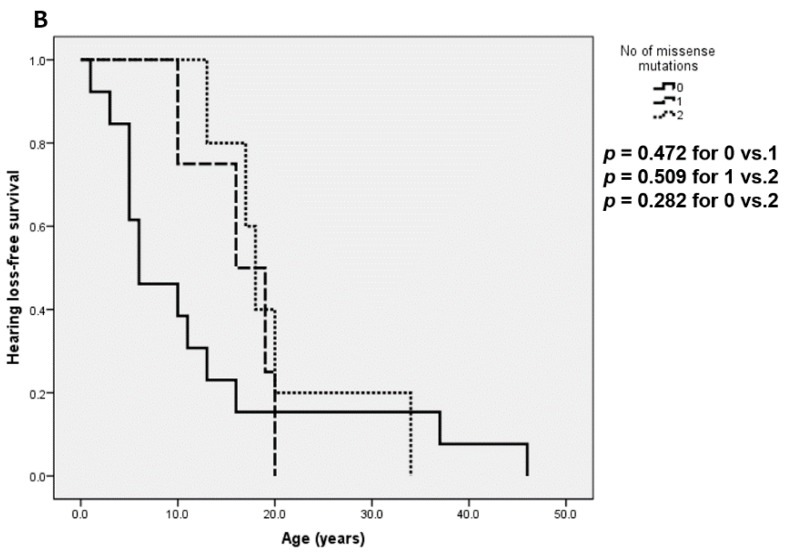
Hearing loss-free survival of ARAS patients according to (**A**) presence of missense mutation and (**B**) number of missense mutations.

We also compared ESRD- and SNHL-free survival between the subgroups of patients (Figure 2). There was a significant deceleration of ESRD-free survival in the group with no missense mutation compared to the group with missense mutation(s) (*p* = 0.024, Figure 2A). This trend was more evident when comparing the groups with 0 missense vs. 2 missenses (*p* = 0.016, Figure 2B). For SNHL-free survival comparison, however, the difference was not statistically significant although a similar pattern was observed (Figure 3).

## 4. Discussion

ARAS is a rare disease and early diagnosis requires clinical suspicion based on its phenotypes.

To the best of authors’ knowledge, this is the first systematic review which has examined the phenotypes and genotypes of ARAS, involving 26 studies with 148 patients. 

In XLAS, it has been reported that patients exhibit proteinuria at a median age of 7 years [1] and more than 90% of XLAS patients develop ESRD by the age of 40 years, with the median age of development of ESRD being 25 years [49]. In female XLAS, 12% of cases develop ESRD by the age of 40 [50] to 65 years [51]. In previous reports of ARAS, the median age at ESRD was 21 years [1,7]. In addition, from the analysis of 15 studies that were available for aggregate patient data (APD), 55% of ARAS patients progressed to ESRD at 23.5 median years (Table 5). In our systematic review of ARAS, the median onset age of proteinuria was 6.5 years, which was comparable to XLAS males. In this study, 53% of the ARAS patients developed ESRD at median of 21 years and this was in line with previous reports [1,7] and the pooled data from APD (Table 5). Regarding pathology, 10% of patients had only diffuse thinning of the GBM and more than 20% of them showed normal type IV collagen α5 expression. Thus, diagnosis of AS based on pathology requires caution, especially in the early disease course. Furthermore, if clinically suspected, confirmation by genetic testing seems more reliable than pathology reports and biopsy may subsidiarily support the diagnosis. 

SNHL has been reported in 50% of male XLAS by 15 years, and 90% by the age of 40 years [1,49,50]. In ARAS, SNHL has been observed at a median age of 20 years [1,7] and the pooled data from APD showed 74% of SNHL at 27 years (Table 5). However, this systematic review showed that 64% of ARAS patients had SNHL at 13 years, implicating that SNHL may present earlier than previously reported.

Ocular abnormalities, including anterior lenticonus, posterior subcapsular cataracts, posterior polymorphous dystrophy, and retinal flecks, are known as typical ophthalmologic complications of AS [1]. From the APD data, ocular manifestation was reported in 66% of ARAS patients at 30 median years (Table 5). In the systematic review, however, the corresponding prevalence was 17% for developing ocular lesions. It was difficult to investigate the age at ocular presentation because only two studies [37,47] provided the relevant information. We postulate that the pooled APD data may have been subject to selection bias because 4 studies [8,9,12,19] were performed on patients (and their family members) who had ocular abnormalities and visited departments of ophthalmology.

Previously, there were two studies by Storey et al. [41] and Oka et al. [7], respectively, which investigated genotype phenotype correlation in ARAS. Storey et al. [41] reported that patients with one or more truncating mutations showed earlier onset of renal failure compared to those without. In contrast, Oka et al. [7] analyzed 30 ARAS patients and reported no genotype phenotype correlation according to the presence of truncating mutations. However, they observed a trend that patients with missense mutations in at least one alleles showed milder phenotypes [7]. Meanwhile, it has been previously shown that the onset age of ESRD in male XLAS patients is significantly delayed in cases possessing missense mutations [1,49,52,53]. In our study, we were unable to group the mutations into truncating vs. non-truncating ones, because most of the splice site variants were not confirmed for sequence changes at the RNA levels. Instead, we grouped the mutations into missense and non-missense ones to make comparisons more easily with previously reported data from XLAS. As a result, we were able to observe a likewise genotype phenotype correlation as in XLAS. The age at both renal and extrarenal manifestations significantly delayed in those with missense mutations. ESRD was more prevalent and had earlier onset of age in patients with no missense mutations. Although it was statistically powerful that patients without missense had higher prevalence of SNHL (*p* < 0.001) and ocular abnormalities (*p* = 0.006), the relevant survival plot was not statistically significant (Figure 3). This inconsistency may be attributed to a paucity of data regarding the age at SNHL and ocular manifestations. Furthermore, Oka et al. [7] reported that some patients with missense mutations had milder renal pathology with full expression of α5 on GBM, and in their study those who had α5 expression on GBM had at least one missense mutation. In our systematic review, patients who had full expression of α5 possessed at least one missense mutation, largely supporting their findings.

In comparison analysis between groups with and without missense mutation(s), we observed that Asian ethnicity was more prevalent in the missense mutation group. The results, however, may be too biased to be generalized. While most of the Caucasian patients were of various origin, such as German, Greek, French, and Australian, Asian patients were either Japanese or Chinese. In addition, there were two large case series consisting 75% of all Asian patients, involving 30 Japanese and 15 Chinese, respectively. The association between ethnicity and having missense mutations requires further investigation.

There are some limitations in our research. Firstly, information retrieved from the available literature may have been exposed to biases. For example, information on SNHL greatly relied upon the clinicians’ reports, which had variable sources: SNHL was sometimes self-reported by the patients and sometimes confirmed by formal tests. Moreover, in cases that received testing, the exact tools or methods of the hearing-ability tests were not specified. A similar problem of inconsistency exists in counting ocular abnormalities. Not all of the patients included in this study may have undergone the same level of ophthalmologic screening. In addition, presence of renal and extrarenal manifestation may have a substantial bias. There were many family cases detected by extended screening from an index patient, and these cases included very young asymptomatic patients, which may have affected the results regarding detection of clinical manifestation. There may have also been potential reporting bias by the patients and clinicians. Secondly, where possible, two pathogenic mutations in the *COL4A3* or *COL4A4* gene on different chromosomes should have been confirmed by testing both parents of the affected individuals as precised in Guidelines [4]. However, although most of the included studies (19 out of 26, 73%) tried to prove in trans mutations, trio testing was not always available for all 148 patients and their parents. Thirdly, analyzing ESRD- and SNHL-free survival plots, there could have been left truncation and survivorship bias for individuals who had more severe genotypes. Fourth, there remained a possibility of existing case reports or series that were not accessible at the time of the literature search. Lastly, results from aggregate patient data (Table 5) may have contained patients who were not genotype proven. Nevertheless, this study has its strengths in that it provides a pooled data and combined evidence on a rare disease. We believe that accumulation of these attempts would contribute to progress. 

## 5. Conclusions

This was a systematic review on genotypes and phenotypes of ARAS involving 26 studies with 148 patients. Patients had ESRD in their early twenties. Hearing loss developed earlier than previously reported. There was genotype phenotype correlation according to the number of missense mutations. Patients with missense mutations had delayed onset of hematuria, ESRD, and SNHL, and a lower prevalence of extrarenal manifestations. Patients who had no missense mutations were more related to consanguinity and had more grave outcomes at earlier age.

## Figures and Tables

**Table 1:** Summary profiles of individual patient data with autosomal recessive Alport syndrome.

Author, Year, Reference	N° Patients ^†^	Sex (M/F)	Ethnicity	Renal Manifestation Frequency (Age in Median Years)					Extrarenal Manifestation Frequency (Age in Median Years)		Age at (Median Years)		Genotypes	
				HU	PU	ESRD	TPL	Initial Pathology	SNHL	Ocular Lesions	Dx of ARAS	Last F-U	Causative Gene	N° Missense Mutations (N° Patients)
Vos, 2018 [23]	2	2/0	n/a	2/2 (2)	n/a	n/a	n/a	1 AS 1 normal	n/a	n/a	n/a	n/a	1 *COL4A3*, 1 *COL4A4*	0 missense (1) 1 missense (1)
Braunish, 2018 [24]	1	0/1	Caucasian	1/1 (12)	1/1 (n/a)	0/1	0/1	FSGS	1/1 (34)	0/1	34	34	1 *COL4A3*	2 missense (1)
Truong, 2017 [25,26]	1	1/0	Caucasian	1/1 (n/a)	1/1 (4.5)	0/1	0/1	Normal	n/a	n/a	n/a	10	1 *COL4A3*	0 missense (1)
Papazachariou, 2017 [27]	7	4/3	Caucasian	7/7 (19)	7/7 (n/a)	2/7 (21)	1/7 (22)	3 FSGS 1TBMD	2/7 (n/a)	n/a	n/a	n/a	7 *COL4A4*	0 missense (2) 2 missense (5)
Liu,2017 [28]	3	n/a	Asian	n/a	n/a	n/a	n/a	n/a	3/3 (n/a)	n/a	n/a	n/a	2 *COL4A3*, 1 *COL4A4*	2 missense (3)
Kamijo, 2017 [29]	1	1/0	Asian	1/1 (n/a)	1/1 (n/a)	0/1	n/a	AS	1/1 (37)	0/1	41	41	1 *COL4A3*	0 missense (1)
Ebner, 2017 [30]	2	2/0	Caucasian	2/2 (0.7)	2/2 (n/a)	0/2	n/a	AS	2/2 (6)	1/2 (n/a)	n/a	19	2 *COL4A3*	0 missense (2)
Uchida, 2016 [31]	4	3/1	Asian	4/4 (n/a)	4/4 (n/a)	2/4 (30)	n/a	AS	1/4 (n/a)	1/4 (n/a)	6.5	24.5	4 *COL4A3*	2 missense (4)
Nishizawa, 2016 [32]	1	0/1	Asian	1/1 (6)	1/1 (23)	n/a	n/a	AS	n/a	n/a	27	n/a	1 *COL4A4*	2 missense (1)
Gast, 2016 [33]	2	1/1	n/a	2/2 (n/a)	n/a	0/2	n/a	2 FSGS	n/a	n/a	33	n/a	2 *COL4A3*	2 missense (2)
Sirisena, 2015 [34]	4	1/3	Caucasian	4/4 (n/a)	4/4 (n/a)	1/4 (15)	n/a	2 MPGN 1FSGS	3/4 (n/a)	0/4	42	n/a	4 *COL4A3*	0 missense (4)
Xie, 2014 [35]	2	2/0	Asian	2/2 (1)	2/2 (19)	n/a	n/a	n/a	2/2 (n/a)	n/a	n/a	n/a	1 *COL4A3*, 1 *COL4A4*	0 missense (1) 2 missense (1)
Webb, 2014 [36]	3	0/3	Caucasian	3/3 (2)	3/3 (2)	n/a	n/a	AS	3/3 (n/a)	0/3	n/a	n/a	3 *COL4A3*	0 missense (3)
Ramzan, 2014 [37]	3	1/2	Caucasian	3/3 (n/a)	3/3 (n/a)	3/3 (20)	1/3 (25)	1 AS	3/3 (7)	2/3 (31)	n/a	31	3 *COL4A4*	0 missense (3)
Oka, 2014 [7]	30	14/16	Asian	30/30 (n/a)	n/a	13/30	n/a	2 TBMD 24 AS	12/30 (14.5)	3/30 (n/a)	18	n/a	23 *COL4A3*, 7 *COL4A3*	0 missense (6) 1 missense (13) 2 missense (11)
Fu, 2014 [38]	1	1/0	Asian	1/1 (2)	1/1 (2)	1/1 (n/a)	1/1 (22)	TBMD	1/1 (17)	0/1	7	n/a	1 *COL4A3*	2 missense (1)
Anazi, 2014 [39]	3	1/2	Caucasian	3/3 (n/a)	3/3 (5.5)	1/3 (12)	2/3 (12)	AS	1/3 (n/a)	0/3	n/a	n/a	3 *COL4A4*	0 missense (3)
Uzak, 2013 [40]	4	3/1	Caucasian	4/4 (n/a)	4.4 (n/a)	4/4 (15)	3/4 (25)	n/a	4/4 (n/a)	4/4 (n/a)	29	n/a	4 *COL4A3*	0 missense (4)
Storey, 2013 [41]	40	19/21	Caucasian ^‡^	40/40 (n/a)	n/a	20/40 (22.5)	n/a	1 TBMD 39 AS	23/40 (n/a)	10/40 (n/a)	31	n/a	20 *COL4A3*, 20 *COL4A4*	0 missense (20) 1 missense (12) 2 missense (8)
Kaimori, 2013 [42]	2	1/1	Asian	2/2 (n/a)	2/2 (n/a)	2/2 (20.5)	2/2 (19.5)	AS	1/2 (5)	1/2 (n/a)	10	n/a	2 *COL4A3*	0 missense (2)
Zhang, 2012 [43]	15	8/7	Asian	15/15 (3.8)	n/a	n/a	n/a	n/a	7/15 (n/a)	1/15 (n/a)	7.5	n/a	13 *COL4A3*, 2 *COL4A4*	0 missense (8) 1 missense (5) 2 missense (2)
Cook, 2008 [44]	2	0/2	African	2/2 (n/a)	2/2 (n/a)	1/2 (14)	1/1 (15)	n/a	2/2 (12)	0/2	12.5	15.5	2 *COL4A3*	0 missense (2)
Rana, 2007 [45]	1	1/0	n/a	1/1 (n/a)	1/1 (n/a)	1/1 (n/a)	n/a	n/a	1/1 (n/a)	1/1 (n/a)	55	55	1 *COL4A3*	0 missense (1)
Hou, 2007 [46]	1	1/0	Asian	1/1 (n/a)	1/1 (n/a)	1/1 (30)	n/a	AS	1/1 (n/a)	1/1 (n/a)	28	n/a	1 *COL4A3*	2 missense (1)
Longo, 2006 [47]	6	2/4	Caucasian	6/6 (7)	6/6 (21)	3/6 (20.5)	3/6 (22)	1 TBMD 2AS	2/6 (27.5)	1/6 (32)	27.5	31	1 *COL4A3*, 5 *COL4A4*	0 missense (2) 2 missense (4)
Vega, 2003 [48]	7	2/5	n/a	7/7 (n/a)	7/7 (n/a)	3/7 (26.5)	n/a	n/a	6/7 (n/a)	2/7 (n/a)	n/a	n/a	6 *COL4A3*, 1 *COL4A4*	0 missense (3) 2 missense (4)

N°, number of; n/a, not available for information; AS, Alport syndrome; ARAS, autosomal recessive Alport syndrome; HU, hematuria; PU, proteinuria; ESRD, end-stage renal disease; TPL, transplantation; F-U, follow up; SNHL, sensorineural hearing loss; FSGS, focal segmental glomerulosclerosis; TBMD, thin basement membrane disease; MPGN, membranoproliferative glomerulonephritis. ^†^ Genotype proven (fulfilling the inclusion criteria of this study) patients only. ^‡^ 21/40 patients were n/a for ethnicity information.

**Table 2:** Features of ARAS patients and comparison of groups with and without missense mutations.

	Total (*n* = 148)	Without Missense (*n* = 69)	With Missense (*n* = 79)	*p*
	N˚ (%)	N˚ (%)	N˚ (%)	Without vs. With
**Causative gene**				
*COL4A3*	96 (65%)	46	50	0.668
*COL4A4*	52 (35%)	23	29	
**Ethnicity**				
Caucasian	53	32/52 (62%)	21/63 (33%)	0.001
Asian	60	18/52 (35%)	42/63 (67%)	
African	2	2/52 (4%)	0/63 (0%)	
n/a	33	17	16	
**Family**				
Consanguineous	31/102 (30%)	27/57 (47%)	6/39 (15%)	<0.001
Positive family Hx	44/57 (77%)	27/35 (77%)	14/22 (63%)	0.991
**Sex**				
Male	71/145 (49%)	36/69 (52%)	35/76 (44%)	0.461
Female	74/145 (51%)	33/69 (48%)	41/76 (52%)	
n/a	3 (2%)	0	3 (4%)	
**Age (median, years)**				
HU	2.5	2.0	5.6	0.004
PU	6.5	3.8	20	0.044
Diagnosis	20	20	19.5	0.231
ESRD	21	19	26	0.006
TPL	20	19	25.5	0.088
SNHL	13	6.5	18	0.019
OT	32 (2 cases)	32 (2 cases)	n/a	-
Last F-U	27	19	27	0.516
**Renal**				
HU	93/93	54/54	39/39	-
PU	89/89	53/53	36/36	-
ESRD	59/95 (62%)	34/48 (71%)	25/47 (54%)	0.076
TPL	14/21 (67%)	12/17 (71%)	2/4 (50%)	0.587
**Extrarenal**				
SNHL	82/129 (64%)	54/62 (87%)	28/67 (42%)	<0.001
OT	15/88 (17%)	12/42 (29%)	3/46 (6%)	0.006
**Outcome**				
Alive	147	69	78	
Death	1	0	1	
n/a	0	0	0	

N°, number of patients; n/a, not available for information and excluded from the tests; Hx, history; AS, Alport syndrome; ARAS, autosomal recessive Alport syndrome; HU, hematuria; PU, proteinuria; ESRD, end-stage renal disease; TPL, transplantation; F-U, follow up; SNHL, sensorineural hearing loss; OT, ocular abnormalities; FSGS, focal segmental glomerulosclerosis; TBMD, thin basement membrane disease; MPGN, membranoproliferative glomerulonephritis.

**Table 3:** Pathology profiles of individual patient data with ARAS.

Study	N˚ patients ^†^	Age at Biopsy (year)	LM	EM	Collagen IV Stain in GBM	Initial–Pathology Diagnosis	Mutation	Zygosity	N˚ Missense
Vos, 2018 [23]	2/2	n/a	Normal	Splitting	α4:(−)	AS	Truncating	H	0
Vos, 2018 [23]		n/a	Normal	Normal	Normal	Normal	Splicing/missense	ch	1
Braunisch, 2018 [24]	1/1	21 (1st) ^‡^ 32 (2nd) ^‡^	Normal (1st) 4/11 GS (2nd)	Normal (1st) lamellation (2nd)	n/a	Nonspecific (1st) → AS (2nd)	Missense	ch	2
Truong, 2017 [25,26]	1/1	4.6	Normal	Normal	Normal	Normal	Duplication	H	0
Papazachariou, 2017 [27]	4/7	n/a	FSGS	n/a	n/a	FSGS	Truncating	H	0
		n/a	FSGS	n/a	n/a	FSGS	Truncating	H	0
		n/a	Normal	Thinning	n/a	TBMD	Missense	H	2
		n/a	FSGS	n/a	n/a	FSGS	Missense	H	2
Liu, 2017 [28]	3/3	15	FSGS	Lamellation	α3:mosaic, α5:mosaic	AS/FSGS	Missense	ch	2
		13	FSGS	Lamellation	α3:mosaic, α5:(−)	AS/FSGS	Missense	H	2
		18		Lamellation	α3:mosaic, α5:mosaic	AS	Missense	H	2
Kamijo, 2017 [29]	1/1	39	GS + SS	Lamellation splitting	α5:(+)	Atypical AS	Splicing	ch	0
Ebner, 2017 [30]	1/2	4	n/a	Irregularity	n/a	AS	Truncating	H	0
Uchida, 2016 [31]	2/4	7	Mesangial proliferation	Lamellation	α5:(−)	AS	Missense	ch	2
		6	Mesangial proliferation	Lamellation	α5:(−)	AS	Missense	ch	2
Nishizawa, 2016 [32]	1/1	27	Normal	Lamellation	α5:reduced	AS	Missense	H	2
Gast, 2016 [33]	2/2	n/a	FSGS	Splitting	n/a	FSGS	Missense	ch	2
		n/a	Normal	n/a	n/a	Normal	Missense	ch	2
Sirisena, 2015 [34]	1/4	14	FSGS	n/a	n/a	FSGS	Truncating	H	0
Xie, 2014 [35]	2/2	n/a	n/a	n/a	α3:(−), α5:(−)	AS	Truncating	H	0
		n/a	n/a	n/a	α3:(−), α5:(−)	AS	Missense	H	2
Webb, 2014 [36]	1/3	3	n/a	Lamellation	n/a	AS	Deletion	H	0
Ramzan, 2014 [37]	1/3	15	n/a	Lamellation	n/a	AS	Truncating	H	0
Oka, 2014 [7]	30/30	n/a	n/a	BWC	α3:(+), α4:(+), α5:(+)	AS	Missense	ch	2
		n/a	n/a	BWC	α5:(+)	AS	Splicing/missense	ch	1
		n/a	n/a	BWC	α5:(−)	AS	Splicing/truncating	ch	0
		n/a	n/a	BWC	α5:(−)	AS	Missense	H	2
		n/a	n/a	BWC	α5:(−)	AS	Truncating	ch	0
		n/a	n/a	BWC	α5:(−)	AS	Missense	ch	2
		n/a	n/a	BWC	n/a	AS	Missense	ch	2
		n/a	n/a	n/a	n/a	AS	Missense	ch	2
		n/a	n/a	BWC	n/a	AS	Missense	ch	2
		n/a	n/a	n/a	n/a	AS	Missense	ch	2
		n/a	n/a	BWC	n/a	AS	Deletion/missense	ch	1
		n/a	n/a	BWC	n/a	AS	Deletion/missense	ch	1
		n/a	n/a	TBMD	α5:(−)	n/a	Missense/truncating	ch	1
		n/a	n/a	BWC	α5:(+)	AS	Missense	H	2
		n/a	n/a	BWC	n/a	AS	Splicing	H	2
		n/a	n/a	n/a	n/a	AS	Splicing	H	2
		n/a	n/a	BWC	α5:(−)	AS	Missense/insertion	ch	1
		n/a	n/a	TBMD	α5:(−)	n/a	Missense/splicing	ch	1
		n/a	n/a	BWC	α5:(−)	AS	Truncating/missense	ch	1
		n/a	n/a	BWC	α5:(−)	AS	Missense/deletion	ch	1
		n/a	n/a	BWC	α5:(−)	AS	Missense	H	2
		n/a	n/a	BWC	α3:(−), α4:(−), α5:(−)	AS	Missense/deletion	ch	1
		n/a	n/a	BWC	α3:(+), α4:(+), α5:(+)	AS	Missense/truncating	ch	1
		n/a	n/a	BWC	n/a	AS	Deletion	H	2
		n/a	n/a	n/a	n/a	AS	deletion	H	2
		n/a	n/a	BWC	α3:(−), α4:(−), α5:(−)	AS	Truncating/missense	ch	1
		n/a	n/a	BWC	α5:(−)	AS	Missense	ch	2
		n/a	n/a	BWC	α5:(−)	AS	Missense/truncating	ch	1
		n/a	n/a	BWC	α5:(−)	AS	Missense	ch	2
		n/a	n/a	BWC	α5:(−)	AS	Missense/deletion	ch	1
Fu, 2014 [38]	1/1	7	n/a	Thinning	α5:(−)	AS	Missense	H	2
Anazi, 2014 [39]	2/3	8.5	n/a	Lamellation	n/a	AS	Truncating	ch	0
		n/a	n/a	Lamellation	n/a	AS	Truncating	ch	0
Hou, 2007 [46]	1/1	26	n/a	Lamellation	α3:(−), α4:(−), α5:(−)	AS	Missense	H	2
Longo, 2006 [47]	3/6	5	n/a	Thinning	n/a	TBMD	Missense	ch	2
		24	n/a	Lamellation	n/a	AS	Missense	H	2
		22	n/a	Splitting	n/a	AS	Missense	H	2
Pooled data	60 (49 EM)	14 median years	7/49 FSGS 7 normal	42/49 AS 5 TBMD 2 normal	23/34 absent α5 3/34 abnormal α5 8/34 normal α5	47/58 AS 5 FSGS 4 normal 2 TBMD			

N°, number of; n/a, not available for information; AS, Alport syndrome; ARAS, autosomal recessive Alport syndrome; HU, hematuria; PU, proteinuria; ESRD, end-stage renal disease; TPL, transplantation; F-U, follow up; SNHL, sensorineural hearing loss; FSGS, focal segmental glomerulosclerosis; TBMD, thin basement membrane disease; MPGN, membranoproliferative glomerulonephritis; GS, global sclerosis; SS, segmental sclerosis; BWC, basket-weave changes; H, homozygous; ch, compound heterozygous. ^†^ Patients available for pathologic report/genotype proven (fulfilling the inclusion criteria of this study) patients. ^‡^ 1st, first kidney biopsy; 2nd, second time kidney biopsy.

**Table 4:** Features of ARAS patients according to the number of missense mutations.

N° Missense Mutations	0 Missense (*n* = 69)	1 Missense (*n* = 31)	2 Missense (*n* = 48)	*p*
	N˚ (%)	N˚ (%)	N˚ (%)	0 vs. 2
**Causative gene**				
*COL4A3*	46	20	30	0.642
*COL4A4*	23	11	18	
**Ethnicity**				
Caucasian	32/52 (62%)	5/23 (22%)	16/40 (40%)	0.002
Asian	18/52 (35%)	18/23 (78%)	24/40 (60%)	
African	2/52 (4%)	0/23 (0%)	0/40 (0%)	
ND	17	8	8	
**Family**				
Consanguineous	27/57 (47%)	0/18 (0%)	6/21 (29%)	0.028
Positive family Hx	27/35 (77%)	3/6 (50%)	14/16 (88%)	0.387
**Sex**				
Male	36 (52%)	14/31 (45%)	21/45 (47%)	0.565
Female	33 (48%)	17/31 (55%)	24/45 (53%)	
n/a	0	0	3	
**Age (median, years)**				
HU	2.0	3.4	10.5	0.005
PU	3.8	n/a	20	0.044
Diagnosis	20	17	20	0.612
ESRD	19	22	30	0.005
TPL	19	n/a	25.5	0.088
SNHL	6.5	17.5	18	0.038
OT	32 (2 cases)	n/a	n/a	-
Last F-U	19	n/a	27	0.516
**Renal**				
HU	54/54	13/13	26/26	-
PU	53/53	n/a	26/26	-
ESRD	34/48 (71%)	11/20 (55%)	14/27 (52%)	0.100
TPL	12/17 (71%)	n/a	2/4 (50%)	0.587
**Extrarenal**				
SNHL	54/62 (87%)	10/25 (40%)	18/42 (43%)	<0.001
OT	12/42 (29%)	2/20 (10%)	1/26 (3%)	0.012
**Outcome**				
Alive	69	31	47	-
Death	0	0	1	
n/a	0	0	0	

N°, number of; n/a, not available for information; Hx, history; AS, Alport syndrome; ARAS, autosomal recessive Alport syndrome; HU, hematuria; PU, proteinuria; ESRD, end-stage renal disease; TPL, transplantation; F-U, follow up; SNHL, sensorineural hearing loss; OT, ocular abnormalities; FSGS, focal segmental glomerulosclerosis; TBMD, thin basement membrane disease; MPGN, membranoproliferative glomerulonephritis.

**Table 5:** Analysis of studies available for aggregate patient data.

Author	N˚ ARAS	ESRD		SNHL		OT	
	N˚	N˚ (%)	Median Age	N˚ (%)	Median Age	N˚ (%)	Median Age
Chen, 2018 ^‡^ [8]	4	n/a	23	3/4 (75%)	n/a	4/4 (100%)	n/a
Savige, 2017 ^‡^ [9]	13	12/13 (92%)	n/a	12/13 (92%)	n/a	13/13 (100%)	n/a
Nabais, 2015 [10]	15	15/15 (100%)	23	9/10 (90%)	32	3/9 (30%)	30
Wang, 2014 [11]	14	n/a	n/a	6/9 (67%)	n/a	0/8 (0%)	n/a
Wang, 2014 ^‡^ [12]	15	14/15 (93%)	27.2	15/15 (100%)	n/a	13/15 (87%)	n/a
Yao, 2012 [13]	24	n/a	n/a	8/24 (30%)	n/a	7/12 (58%)	n/a
Temme, 2012 [14]	29	3/29	n/a	n/a	n/a	n/a	n/a
Artuso, 2012 [15]	2	1/2 (50)	n/a	n/a	n/a	n/a	n/a
Pierides, 2009 [16]	42	18/42 (43%)	n/a	n/a	n/a	n/a	n/a
Shaw, 2007 [17]	7	6/6 (100%)	25	7/7 (100%)	32	7/7 (100%)	32
Wei, 2006 [18]	13	3/13 (23%)	17	6/12 (50%)	22	3/12 (25%)	26
Dagher, 2002 ^‡^ [19]	11	8/11 (72%)	24	10/11 (91%)	n/a	10/11 (91%)	n/a
Heidet, 2000 [20]	60	44/60 (73%)	22	27/35 (77%)	n/a	16/26 (62%)	n/a
Torra, 1999 [21]	5	2/5 (40%)	33	4/5 (80%)	13.5	0/5 (0%)	n/a
Boye, 1998 [22]	10	8/31 (26%)	n/a	n/a	n/a	n/a	n/a
Pooled data	264	134/242 (55%)	23.5	107/145 (74%)	27	76/115 (66%)	30

n/a: not available for information; ESRD, end-stage renal disease; SNHL, sensorineural hearing loss; OT, ocular abnormalities. ^‡^ These studies were performed on patients (and their family members) who had ocular abnormalities and visited department of ophthalmology.

## Supplementary Materials

The following are available online at http://www.mdpi.com/2077-0383/8/2/178/s1, Table S1: PRISMA 2009 Checklist.
